# Self‐reported navigation ability is associated with optic flow‐sensitive regions’ functional connectivity patterns during visual path integration

**DOI:** 10.1002/brb3.1236

**Published:** 2019-03-18

**Authors:** Lauren Zajac, Heather Burte, Holly A. Taylor, Ronald Killiany

**Affiliations:** ^1^ Department of Anatomy & Neurobiology Boston University School of Medicine Boston Massachusetts; ^2^ Center for Biomedical Imaging Boston University School of Medicine Boston Massachusetts; ^3^ Department of Psychology Tufts University Medford Massachusetts

**Keywords:** fMRI, humans, optic flow, parietal lobe, spatial ability, spatial navigation

## Abstract

**Introduction:**

Spatial navigation is a complex cognitive skill that varies between individuals, and the mechanisms underlying this variability are not clear. Studying simpler components of spatial navigation may help illuminate factors that contribute to variation in this complex skill; path integration is one such component. Optic flow provides self‐motion information while moving through an environment and is sufficient for path integration. This study aims to investigate whether self‐reported navigation ability is related to information transfer between optic flow‐sensitive (OF‐sensitive) cortical regions and regions important to navigation during environmental spatial tasks.

**Methods:**

Functional magnetic resonance imaging was used to define OF‐sensitive regions and map their functional connectivity (FC) with the retrosplenial cortex and hippocampus during visual path integration (VPI) and turn counting (TC) tasks. Both tasks presented visual self‐motion through a real‐world environment. Correlations predicting a positive association between self‐reported navigation ability (measured with the Santa Barbara Sense of Direction scale) and FC strength between OF‐sensitive regions and retrosplenial cortex and OF‐sensitive regions and the hippocampus were performed.

**Results:**

During VPI, FC strength between left cingulate sulcus visual area (L CSv) and right retrosplenial cortex and L CSv and right hippocampus was positively associated with self‐reported navigation ability. FC strength between right cingulate sulcus visual area (R CSv) and right retrosplenial cortex during VPI was also positively associated with self‐reported navigation ability. These relationships were specific to VPI, and whole‐brain exploratory analyses corroborated these results.

**Conclusions:**

These findings support the hypothesis that perceived spatial navigation ability is associated with communication strength between OF‐sensitive and navigationally relevant regions during visual path integration, which may represent the transformation accuracy of visual motion information into internal spatial representations. More broadly, these results illuminate underlying mechanisms that may explain some variability in spatial navigation ability.

## 1 INTRODUCTION

Spatial navigation is a complex, multisensory process that fundamentally requires understanding and updating one's location within large‐scale space (Wolbers & Hegarty, 2010). A large amount of natural variability in environmental spatial ability exists among individuals (Arnold et al., 2013; Weisberg, Schinazi, Newcombe, Shipley, & Epstein, 2014), and breaking this complex skill into component processes is necessary to unravel the mechanisms underlying this natural variability (Chrastil, 2013). One central aspect of spatial navigation is dead reckoning or path integration, which is the use of self‐motion cues to keep track of one's location and orientation in space (Etienne & Jeffery, 2004; Loomis, Klatzky, Golledge, & Philbeck, 1999). Though the detailed relationship between path integration ability and humans’ ability to navigate within their environment is not precisely understood, path integration is thought to be associated with the construction of survey‐level representations of environments, which are the most flexible representations of space in the brain (Arnold et al., 2013; Chrastil, 2013; Etienne & Jeffery, 2004; McNaughton, Battaglia, Jensen, Moser, & Moser, 2006). Gaining a better understanding of how the human brain supports path integration will ultimately allow us to improve our understanding of how we actively navigate within our environment as well as how we build mental representations of space.

Though path integration is a simpler component of the broader and more complex process that is spatial navigation, it is a complex process in itself. It can be achieved using one or more sources of sensory information as well as different strategies. Humans can use sensory information from multiple sources to both estimate self‐motion and path integrate (Kearns, Warren, Duchon, & Tarr, 2002; Klatzky, Loomis, Beall, Chance, & Golledge, 1998; Loomis et al., 1993, 1999; Philbeck, Klatzky, Behrmann, Loomis, & Goodridge, 2001; Tcheang, Bulthoff, & Burgess, 2011) and they may rely on different strategies and/or neural systems to do so (He & McNamara, 2018; Philbeck, Behrmann, Levy, Potolicchio, & Caputy, 2004; Shrager, Kirwan, & Squire, 2008; Wiener, Berthoz, & Wolbers, 2011; Worsley et al., 2001). Due to the complexity created by using multiple sensory sources, path integration tasks have frequently been designed to assess the contribution of different sources of self‐motion information to this process. Self‐motion cues are typically divided into *allothetic cues*, which are sensed from the external environment, or *idiothetic cues*, which are internally generated. Visual and acoustic inputs are allothetic cues, while vestibular signals, proprioceptive afferents, and motor efference copy are idiothetic cues. One important visual self‐motion cue used to path integrate is optic flow, which refers to the pattern of motion on the retina experienced while moving through the environment. While optic flow is not necessary for humans to path integrate, it is sufficient (Ellmore & McNaughton, 2004; Harris & Wolbers, 2012; Kearns et al., 2002; Riecke, Veen, & Bülthoff, 2002; Wiener & Mallot, 2006). In other words, in the absence of idiothetic cues, humans can path integrate with varying levels of accuracy using optic flow alone. The converse is also true—humans can path integrate using only idiothetic cues (Allen, Kirasic, Rashotte, & Haun, 2004; Loomis et al., 1993). Thus, humans are flexible in what sensory information they are capable of using to path integrate, and sensory information may differentially contribute to or dominate this process in different contexts (Allen et al., 2004; Campos, Butler, & Bülthoff, 2012; Chance, Gaunet, Beall, & Loomis, 1998; Kearns et al., 2002; Péruch, Borel, Magnan, & Lacour, 2005; Philbeck et al., 2001). In one example of this, optic flow has been shown to significantly affect individuals’ internal representation of paths even in the presence of idiothetic cues that may not align with the experienced optic flow (Tcheang et al., 2011). This reveals the impact of optic flow on the formation of mental representations of space and aligns with the central role of vision in human spatial navigation (Ekstrom, 2015). Thus, although path integration is a simpler component of spatial navigation—it is a complex process in which sensory information interacts to create an internal representation of space. Restricting sensory information to one modality has been a helpful way to simplify the study of path integration.

Humans’ ability to path integrate using optic flow alone has allowed for the study of neural systems that support this ability using functional magnetic resonance imaging (fMRI). In fMRI studies of path integration—and other types of spatial navigation—participants must perform the task while lying still in the MRI scanner, which limits the sensory input used to perform these tasks to visual information. FMRI studies of visual path integration have largely focused on how brain activity levels relate to performance accuracy during these tasks (Chrastil, Sherrill, Hasselmo, & Stern, 2015, 2016; Sherrill et al., 2013; Wolbers, Wiener, Mallot, & Buchel, 2007). Increased activity in the hippocampus (Chrastil, Sherrill, Hasselmo, & Stern, 2015; Sherrill et al., 2013; Wolbers et al., 2007), retrosplenial cortex (Chrastil et al., 2015; Chrastil, Sherrill, Hasselmo, & Stern, 2016), and prefrontal cortex (Chrastil et al., 2015; Sherrill et al., 2013; Wolbers et al., 2007) has been associated with more accurate path integration performance, while increased activity in the human motion complex hMT+ has been associated with less accurate path integration performance (Wolbers et al., 2007), suggesting that the neural response to visual motion is inversely related to path integration ability. In line with many of these findings, Chrastil et al. (2017) found that gray matter volume in the hippocampus, retrosplenial cortex, and medial prefrontal cortex are positively associated with path integration accuracy in young adults. These studies largely fit in with diverse navigation studies that repeatedly show the importance of the hippocampus and retrosplenial cortex, among others, to navigation performance (see Epstein, Patai, Julian, & Spiers, 2017 for review).

While brain activity levels highlight regions that may be particularly important to certain cognitive skills, in recent years focus has shifted to examining how the interactions between brain regions during tasks or at rest may explain individual variability in cognitive abilities. Emerging models of the way the human brain supports spatial navigation have specifically highlighted these ideas (Ekstrom, Huffman, & Starrett, 2017). Through this lens, interactions between certain brain regions may facilitate performance of some cognitive tasks or relate to perception of one's ability in that domain. Only a few studies have examined this in the context of path integration or spatial navigation in general. Arnold, Burles, Bray, Levy, and Iaria (2014) examined this in the context of visual path integration and found that individuals with stronger interactions among frontal and parietal areas performed more accurately. More recently, stronger interaction between right posterior hippocampus and right retrosplenial cortex at rest (Sulpizio, Boccia, Guariglia, & Galati, 2016) and greater betweenness centrality of right retrosplenial cortex within a network of regions involved in navigation tasks (Kong et al., 2017) at rest were found to be associated with better self‐reported navigation ability. These studies show that task‐related communication between brain regions, as well as their interactions at rest, hold information about the variation in spatial navigation ability between individuals.

In addition to using fMRI to study path integration, fMRI has also been used to study numerous brain regions that respond to and are selective for optic flow, but much of this work has been outside the context of navigation (Braddick et al., 2001; Cardin & Smith, 2009; Morrone et al., 2000; Pitzalis et al., 2013; Wall & Smith, 2008). Recently, Sherrill et al. (2015) connected these two areas of research through measuring the functional connectivity patterns of optic‐flow sensitive (OF‐sensitive) brain regions during a goal‐directed navigation task. They found that OF‐sensitive brain regions increased their interaction with the hippocampus, retrosplenial cortex, and posterior parietal cortex, among other regions, while participants navigated toward a goal in first person perspective. Increased interactions between OF‐sensitive regions and this set of navigationally relevant brain regions were largely absent during a survey‐perspective navigation condition. The communication between relevant sensory regions and navigation regions during navigation tasks has received little attention. In particular, whether the interactions between OF‐sensitive brain regions and navigationally relevant brain regions, such as retrosplenial cortex and hippocampus, are related to spatial navigation ability has not been explored.

To this end, our main objective in this study was to expand upon the findings of Sherrill et al. (2015) by testing the hypothesis that the strength of communication between OF‐sensitive brain regions and navigationally relevant brain regions during visual path integration is positively associated with perceived spatial navigation ability due to better information transfer between these regions in navigational contexts. We designed visual path integration (VPI) and turn counting (TC) tasks employing real‐world stimuli to test this hypothesis. In both tasks, participants viewed videos of short paths filmed in first person perspective. These resembled what they would see if walking down the street in a Boston neighborhood. In the VPI condition, participants needed to use visual motion to keep track of their position and orientation throughout the short paths. They did not control their movement through the environment. This design choice was made to limit, to the extent possible, the cognitive process captured to the use of visual self‐motion to keep track of one's position and orientation relative to a goal location. In TC, participants viewed the same type of stimuli but counted turns, thus navigational demands beyond perceptual processing of the stimuli were not present. A dot‐field task was used to define a set of OF‐sensitive cortical regions using fMRI, and regions previously shown to respond more strongly to motion consistent with self‐motion (or egomotion) (Cardin & Smith, 2009) were selected for our functional connectivity analyses. Psychophysiological interactions (PPI) analyses (Friston et al., 1997; O'Reilly, Woolrich, Behrens, Smith, & Johansen‐Berg, 2012) were used to measure functional connectivity strength between these OF‐sensitive regions and the hippocampus and retrosplenial cortex during VPI and TC. We measured overall self‐reported spatial navigation ability with the Santa Barbara Sense of Direction (SBSoD) scale (Hegarty, Richardson, Montello, Lovelace, & Subbiah, 2002). In contrast to studies that have used task performance as a measure of spatial navigation ability, we wanted to avoid performance as a confounding factor in interpreting the task‐related functional connectivity strength between brain regions in this experiment. Rather, highly accurate task performance allowed us to capture the neural systems to which participants defaulted when performing everyday navigational tasks, and a measure of perceived overall navigation ability allowed us to explore elements of these systems that may be advantageous. Conveniently, humans have been shown to accurately assess their own sense of direction (Hegarty et al., 2002; Kozlowski & Bryant, 1977; Sholl, 1988; Sholl, Kenny, & DellaPorta, 2006) and for this reason, the SBSoD scale has been widely used in the literature.

As a direct test of our hypothesis, we examined the relationship between self‐reported navigation ability and functional connectivity strength between OF‐sensitive regions and the hippocampus and retrosplenial cortex during VPI and assessed the specificity of these relationships to VPI. In a set of whole‐brain exploratory analyses, we investigated whether there was an effect of self‐reported navigation ability on task‐related functional connectivity strength between OF‐sensitive regions and any other brain regions during VPI and TC. This allowed us to explore the potentially efficient and inefficient ways that OF‐sensitive regions interact with the rest of the brain while visually moving through the environment in the presence or absence of navigational demands.

## 2 MATERIALS AND METHODS

### 2.1 Participants

Participants (*n* = 15, 7 female, 14 right‐handed) were recruited from the greater Boston area and had a mean age of 27.1 years (*SD* = 2.66, range = 24–34). No participants had untreated physical or mental disorders. Two participants were on maintenance doses of Adderall, one participant was on a maintenance dose of Fluoxetine, and one participant was on a maintenance dose of Paroxetine. None of these participants reported symptoms related to conditions that these medications are meant to treat. The study was approved by the Institutional Review Board at the Boston University School of Medicine and was conducted in accordance with the Declaration of Helsinki. All participants gave written informed consent acknowledging their participation in this study.

### 2.2 Experimental design

After consent and prior to entering the MRI scanner, instructions for each task were explained. Participants were shown 10 practice trials of the visual path integration (VPI) task (two zero‐, four one‐, and four two‐turn trials, in that order) and four trials of the TC task. They were given the opportunity to ask questions and were given performance feedback during these practice trials. Once positioned in the MRI scanner, they were shown an additional 10 practice trials of the VPI task (two zero‐, four one‐, and four two‐turn trials) to practice performing the task while lying in a supine position and to become familiar with responding with the hand‐held device.

#### 2.2.1 Visual path integration task

**Figure 1:**
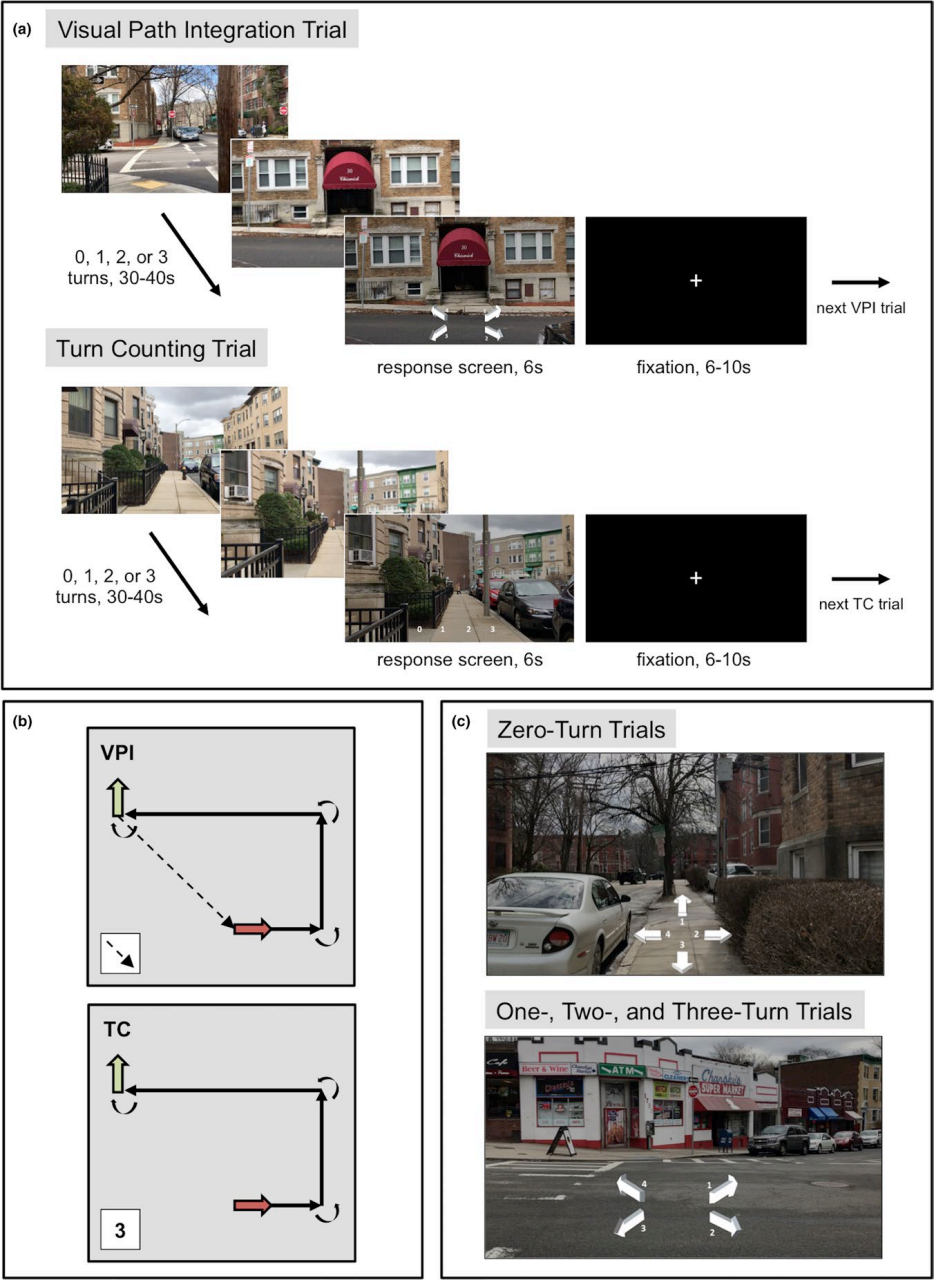
Visual path integration and turn counting tasks. (a) Configuration of VPI and TC trials. Each trial consisted of a 30–40‐s video and a 6‐s response screen. Stills from a VPI and a TC trial are displayed, showing what one would see if he or she were walking that path in Brighton, Massachusetts. The last frame of each trial served as a backdrop for either response arrows (VPI trials) or response numbers (TC trials), corresponding to options for the direction of the start location or number of turns in that trial, respectively. Participants were shown eight trials of either the VPI or TC task per run. A fixation cross was displayed between each trial and at the start and end of each run. (b) “Birds‐eye view” of a three‐turn trial and the correct answer for VPI (top) and TC (bottom). The maroon arrow represents starting location and facing direction and the green arrow represents ending location and facing direction. Participants were required to keep track of starting location in VPI trials. (top) In this VPI trial, the correct answer would be arrow #2 (c, bottom panel), which points to the back and right of the participant's ending location and facing direction. (bottom) In this TC trial, the correct answer would be three because three turns were completed in the path. Participants were not required to keep track of location at all during the TC task. (c) Example of response arrow configuration for zero‐turn trials (top) vs. one‐, two‐, and three‐turn trials (bottom). The same numbers corresponded to the same arrows across trials. TC: turn counting; VPI: visual path integration

In the VPI task, participants used visual stimuli that one would typically experience walking along a short path to keep track of their position and orientation relative to their starting location in each video. The VPI task was composed of a series of short videos (30–40 s) filmed from a first person perspective while walking through a Boston neighborhood. Videos were filmed by the same person (LZ) on the same day using an iPhone 7 at a resolution of 1920 × 1080 pixels at 30 frames/second and a walking pace of 1.7 steps/second, which was maintained with a metronome. Specifically, the individual filming these videos timed her steps according to the metronome, which was set at a speed of 103 beats/minute. All sound was removed from these videos. Each trial (i.e., video) showed a path with zero, one, two, or three 90° turns (Figure 1). The VPI task was composed of eight trials of each type (i.e., zero, one, two, or three turns), which totaled 32 trials. All paths were unique in that none were presented to the participant more than one time. At the end of each VPI trial, four arrows appeared on the dimmed last frame of the video (Figure 1). Participants were asked to select the arrow that pointed to their location at the start of the path relative to their current location and facing direction at the end of the video. They had 6 s to respond, using an MR‐compatible device, with the number corresponding to the arrow that they believed was pointing toward the starting location of the path. Responses were balanced within each trial type across response options. For example, for two‐turn trials, the response could be any one of the four presented arrows, thus the correct responses to this trial type were balanced across all four arrows. For one‐turn trials, the correct response could only be arrow 2 or 3, thus the correct responses were balanced across these two arrows. Between trials, a white fixation cross on a black background appeared for 6, 8, or 10 s.

#### 2.2.2 Turn counting task

After the VPI task, participants completed the TC task, which included 32 different videos with zero, one, two, or three 90° turns, just as in the VPI task. There were eight trials of each type and trials were balanced across runs. The videos presented in each trial were of different paths than those presented in the VPI task, but they were filmed on the same day, in the same neighborhood, by the same person, and with the same parameters as the VPI videos. Again, no path was presented more than once. At the end of each TC trial, 0, 1, 2, and 3 appeared and participants selected the number of turns they believed were made in the video (Figure 1). Thus, the VPI and TC tasks were composed of the same type of stimuli, but the way participants used the visual information differed. The VPI task had navigational demands, but the TC task did not; participants were not required to track their location at all during the TC task.

Participants were shown the same set of stimuli (64 videos total) with individual trials within each task presented in two different orders. During scanning, all participants were first shown four runs of the VPI task and were then shown four runs of the TC task. We did not interleave VPI and TC trial types or task runs because we wanted to minimize confusion over which task was being performed in order to minimize spontaneous path integration during the TC runs. Additionally, our hypothesis was focused on the functional connectivity strength between OF‐sensitive regions and the hippocampus and retrosplenial cortex during VPI, thus all participants completed the VPI task first, immediately after the practice trials.

#### 2.2.3 Optic flow localizer task

A paradigm consisting of alternating blocks of coherent and scrambled motion (Pitzalis et al., 2009, 2013) was used to define OF‐sensitive regions of interest (ROIs). The paradigm was shared with us by Dr. Marty Sereno at SDSU and is freely available (contact msereno@sdsu.edu). The paradigm consists of white dots on a black background that move in alternating 16 s‐blocks of coherent motion (dilation, contraction, inward spiral, outward spiral, or rotation) or scrambled motion. In both coherent and scrambled motion blocks, a new field of white dots appears every 500 ms. The type of coherent motion chosen for each 500 ms period was randomly selected from a continuum. In scrambled blocks, the trajectory of each dot was rotated by a random angle, which disrupted the coherent motion of the dot field. A speed gradient was present such that central dots moved more slowly than peripheral dots in both coherent and scrambled blocks. Each block was shown eight times, starting with a coherent block. A red fixation cross was present in the center of the screen throughout and participants were instructed to stay awake and fixate on the cross throughout four runs of this task.

### 2.3 Magnetic resonance imaging

All participants were scanned at the Center for Biomedical Imaging at the Boston University School of Medicine on a 3T Philips Achieva system with a 32‐channel head coil. The scanning session included the following scans in this order: four VPI runs, four TC runs, four optic flow localizer runs, and a T1‐weighted (T1W) anatomical scan. Axial T2*‐weighted scans with blood‐oxygenation‐level‐dependent (BOLD) contrast were acquired during the VPI, TC, and optic flow localizer runs (TR/TE: 2,000/28 ms, acquired and reconstructed voxel size: 3 × 3 × 3 mm^3^, matrix: 64 × 64, 36 slices, EPI factor: 47). Four dummy scans were acquired at the start of each run. For the VPI and TC runs, the number of dynamics varied with the length of each run (187–193 dynamics). Each optic flow localizer run consisted of 132 dynamics. A sagittal MP‐RAGE scan was used to acquire T1W data (TR/TE: 6.7/3.1 ms, flip angle: 9°, acquired voxel size: 1.11 × 1.11 × 1.2 mm^3^, acquired matrix: 244 × 227, reconstructed voxel size: 1.05 × 1.05 × 1.2 mm^3^, reconstructed matrix: 256 × 256, 140 slices).

The visual stimuli were displayed on an MR‐compatible LCD screen (Cambridge Research Systems, BOLDscreen 3D LCD for fMRI, active area: 50.9 × 29.0 cm^2^) and viewed through a mirror (15.2 × 7.6 cm^2^) mounted on the head coil that was approximately 13 cm from the participants’ eyes and 102 cm from the LCD screen. VPI and TC runs were presented in EPrime v2.0 on a PC running Windows 7 Professional. The optic flow localizer was presented on a MacBook Pro (Retina, 13‐inch, Early 2015, running Yosemite v10.10.5). Lights in the scanner room were dimmed during the experiment. Participants responded to each VPI and TC trial with their dominant hand using a Current Designs PYKA response pad in the scanner.

### 2.4 Santa Barbara Sense of Direction scale

Participants filled out the Santa Barbara Sense of Direction (SBSoD) scale (Hegarty et al., 2002) after the completion of scanning. The SBSoD is a widely used self‐report measure of environmental spatial ability that has been shown to correlate with performance on tests of the ability to update one's position and orientation in space (Arnold et al., 2013; Hegarty et al., 2002). The SBSoD is increasingly being used as a measure of overall spatial navigation ability—especially in MRI studies (Auger, Mullally, & Maguire, 2012; Epstein, Higgins, & Thompson‐Schill, 2005; Halko, Connors, Sánchez, & Merabet, 2014; Kong et al., 2017; Sulpizio et al., 2016; Wegman & Janzen, 2011). The scale is composed of 15 questions to which participants respond on a Likert‐type scale of 1–7 (strongly agree‐strongly disagree). Half of the questions are positively framed (i.e., “I am very good at reading maps) and half are negatively framed (i.e., “It's not important to me to know where I am”). Positive items are reversed‐scored and then the average across all 15 items is calculated. Possible scores range from 1–7 with seven representing strong self‐reported, or subjective, spatial navigation ability.

### 2.5 Image processing

All data were exported from the MRI scanner in FSL‐NIfTI format and visually inspected prior to use. FMRI data were processed in FSL (FMRIB Software Library) v5.0.8 (Jenkinson, Beckmann, Behrens, Woolrich, & Smith, 2012) using the FMRI Expert Analysis Tool (FEAT) v6.00. FEAT uses general linear modeling to fit an experimenter‐specified model of the BOLD signal in each voxel.

#### 2.5.1 Preprocessing

Motion correction with MCFLIRT, spatial smoothing with a FWHM of 6 mm, and high‐pass temporal filtering with a cutoff of 90 s were carried out. Runs were excluded if there were relative motion spikes greater than or equal to 3 mm. Three runs from two participants satisfied this criterion and were excluded from further analysis (one optic flow localizer run from participant one and one VPI and one TC run from participant two). FMRI data were linearly registered to each participant's T1W image using boundary‐based registration and then to the MNI152 2 mm atlas using 12 *df* (Greve & Fischl, 2009; Jenkinson, Bannister, Brady, & Smith, 2002; Jenkinson & Smith, 2001). Each participant's image registration was visually inspected for accuracy.

#### 2.5.2 Defining OF‐sensitive ROIs

Many cortical regions in the human brain have been reported to be responsive to motion and have different degrees of selectivity for different types of motion (for examples see Braddick et al., 2001; Cardin & Smith, 2009; Pitzalis et al., 2013; Wall & Smith, 2008). In an effort to focus our analyses and minimize the number of statistical tests performed, we defined and selected OF‐sensitive ROIs using two criteria. First, we defined a set of regions that responded more strongly to coherent dot motion relative to scrambled dot motion at the group level. To do this, the preprocessed data collected during the optic flow localizer were prewhitened. A double‐gamma HRF convolution was applied to the stimulus waveform representing coherent dot motion; this modeled where brain activity was increased during coherent dot motion compared to scrambled dot motion. The temporal derivative of this model was included in the design matrix. Fixed‐effects analyses were used for within‐subject higher level analyses to determine the average increase in brain activity during coherent dot motion relative to scrambled dot motion across optic flow runs. We then created leave‐one‐out maps to define stable OF‐sensitive ROIs. Leave‐one‐out maps are used to protect against the possibility that the definition of any region is primarily driven by one participant's data. To create these maps, FLAME 1 + 2 was used to create a group average of 14/15 participants’ maps of brain activity using cluster thresholding with a Z threshold of 2.3 and a FWER‐corrected cluster p threshold of 0.05. This was repeated, leaving out each participant, until a total of 15 maps were created. These statistical maps were binarized, summed, and overlaid on the group map of brain activity of all 15 participants (also created with FLAME 1 + 2 using a Z threshold of 2.3 and a FWER‐corrected cluster p threshold of 0.05). Voxels in which an overlap of all 15 leave‐one‐out maps coincided with a peak in brain activity in the 15‐participant group map were selected as the center coordinates of OF‐sensitive ROIs. Spheres with a radius of 5 mm were centered at these coordinates in MNI152 2 mm space.

**Figure 2:**
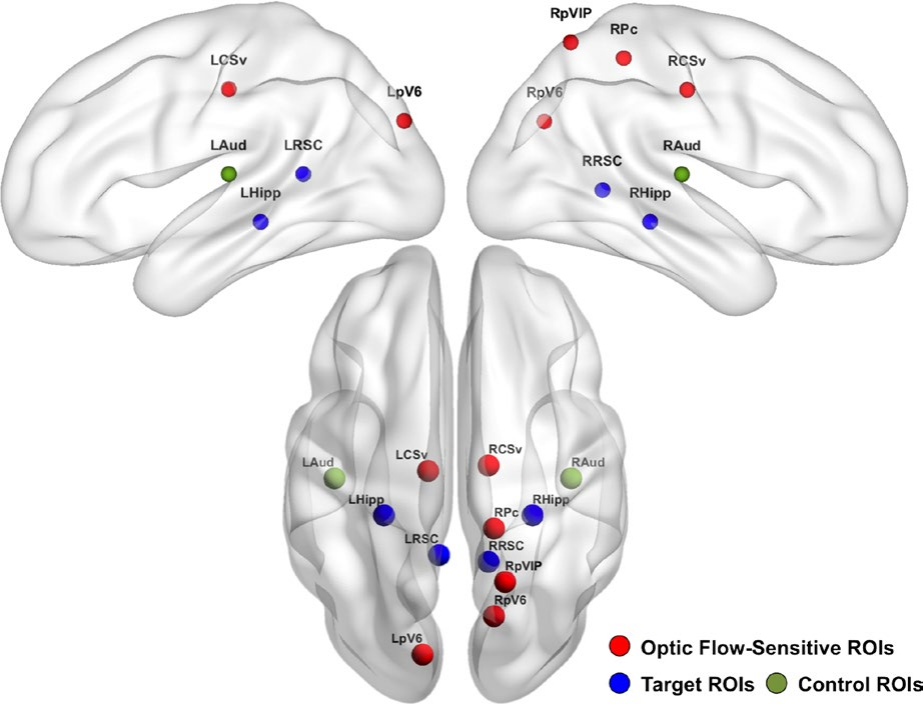
Optic flow‐sensitive, navigationally relevant target, and control regions of interest. The OF‐sensitive (red), navigationally relevant target (blue), and control (green) regions of interest (ROIs) are shown on cortical surface representations created in BrainNet Viewer (Xia, Wang, & He, 2013). The center coordinates of these ROIs are listed in Table 1 in MNI152 space. The OF‐sensitive (L/R CSv, L/R pV6, R Pc, R pVIP), navigationally relevant target (L/R hippocampus (L/R Hipp) and L/R retrosplenial cortex (L/R RSC)), and control (L/R primary auditory cortex (L/R Aud)) ROIs were used in the ROI‐based analyses performed to test our hypothesis, and the OF‐sensitive regions were used as seed regions in our whole‐brain exploratory analyses. L Aud: left primary auditory cortex; L CSv: left cingulate sulcus visual area; L Hipp: left hippocampus; L pV6: left putative area V6; L RSC: left retrosplenial cortex; MNI: Montreal Neurological Institute; OF: optic flow; R Aud: right primary auditory cortex; R CSv: right cingulate sulcus visual area; R Hipp: right hippocampus; ROI: region of interest; R Pc: right precuneus; R pVIP: right putative ventral intraparietal area; R pV6: right putative area V6; R RSC: right retrosplenial cortex

Second, we selected a subset of these regions to test our hypothesis based on their proximity to regions sensitive to “egomotion‐compatible” stimuli reported by Cardin and Smith (2009). In this paper, the authors investigated response selectivity of several cortical visual motion areas to egomotion‐compatible stimuli, or stimuli containing motion compatible with the observer's movement. They identified several areas that were sensitive to egomotion using two conditions of a dot‐field task. The egomotion‐compatible condition contained coherent radial dot motion originating from a single, central focus of expansion. The egomotion‐incompatible condition contained the same forms of coherent radial dot motion arranged in nine patches, which created an array of nine foci of expansion. A comparison of where brain activity was greater during egomotion‐compatible compared to egomotion‐incompatible stimuli allowed the authors to identify cortical areas that were sensitive to egomotion‐compatible stimuli and determine how sensitive they were. We reasoned that regions that responded more strongly to egomotion‐compatible stimuli would be most relevant to our analyses due to our focus on using visual self‐motion cues to path integrate. To identify the regions identified by our optic flow localizer that corresponded to the egomotion‐sensitive regions reported in Cardin and Smith (2009), we measured the Euclidean distance between the center coordinates of Cardin and Smith's regions and the center coordinates of our OF‐sensitive regions. OF‐sensitive regions whose center coordinates were within 14 mm of any of Cardin and Smith's egomotion‐compatible regions were selected for further analysis and named according to the region to which they were closest. This resulted in our selection of the following six OF‐sensitive regions for our analyses: left and right putative V6 (L pV6 and R pV6), left and right cingulate sulcus visual area (L CSv and R CSv), a region in the right precuneus (R Pc), and right putative ventral intraparietal area (R pVIP) (Cardin & Smith, 2009; Wall & Smith, 2008). The center coordinates of these regions are listed in Table 1, and the regions themselves are shown in Figure 2.

**Table 1:** Optic flow‐sensitive, navigationally relevant target, and control regions of interest

Region	ROI type	MNI152 coordinates (*x*, *y*, *z*)
L pV6	OF‐sensitive	−12, −86, 30
L CSv	OF‐sensitive	−10, −20, 42
R pV6	OF‐sensitive	14, −72, 30
R Pc	OF‐sensitive	14, −42, 54
R CSv	OF‐sensitive	12, −18, 42
R pVIP	OF‐sensitive	18, −62, 60
Left Retrosplenial Cortex (L RSC)	Navigation	−6, −48, 10
Right Retrosplenial Cortex (R RSC)	Navigation	10, −48, 8
Left Hippocampus (L Hipp)	Navigation	−26, −32, −8
Right Hippocampus (R Hipp)	Navigation	28, −32, −8
Left Primary Auditory Cortex (L Aud)	Control	−44, −20, 10
Right Primary Auditory Cortex (R Aud)	Control	42, −20, 10

Note

A dot‐field task, leave‐one‐out analysis, and systematic comparison to egomotion‐sensitive regions from Cardin and Smith (2009) were used to define OF‐sensitive regions at the group level in this study. We defined six OF‐sensitive regions using this approach. Spherical, binary ROIs with 5 mm radii were centered at the MNI152 coordinates listed in this table. Navigationally relevant target regions were defined according to a combination of a priori hypotheses about brain regions relevant to spatial navigation and peaks of increased brain activity during VPI relative to rest measured at the group level. Control regions were defined according to a combination of an a priori hypothesis about brain regions that would not be particularly relevant to these tasks and anatomical information in the Harvard‐Oxford Cortical Structural Atlas included in FSLview. Spherical, binary ROIs with a radius of 5 mm were centered at the MNI152 coordinates listed in this table to define navigationally relevant target and control regions. Figure 2 shows a visual representation of these ROIs.

L Aud: left primary auditory cortex; L CSv: left cingulate sulcus visual area; L Hipp: left hippocampus; L pV6: left putative area V6; L RSC: left retrosplenial cortex; MNI: Montreal Neurological Institute; OF: optic flow; R Aud: right primary auditory cortex; R CSv: right cingulate sulcus visual area; R Hipp: right hippocampus; ROI: region of interest; R Pc: right precuneus; R pVIP: right putative ventral intraparietal area; R pV6: right putative area V6; R RSC: right retrosplenial cortex.

#### 2.5.3 Defining target and control ROIs

The hypothesis we set out to test is that stronger communication between OF‐sensitive regions and brain regions important to navigation during VPI is positively associated with self‐reported spatial navigation ability. This required us to select target ROIs that are relevant and important to spatial cognition (target ROIs) as well as regions that should not be relevant (control ROIs). The hippocampus and retrosplenial cortex have been shown to be important to spatial navigation processes in numerous studies, thus we selected these regions as navigationally relevant target ROIs. However, because different regions of these structures have been shown to be relevant to different aspects of spatial navigation across different MRI studies, we defined spherical ROIs relevant to our VPI task within each of these anatomical regions in the right and left hemispheres. The center coordinates of these ROIs were defined using a group‐level brain activation map showing significantly increased brain activity during VPI relative to rest. Peaks of increased brain activity within significant clusters in each of these regions were selected as the center coordinates of these ROIs, and binary spheres with a radius of 5 mm were centered at these coordinates. These coordinates are listed in Table 1 and the ROIs are shown in Figure 2. The right and left hippocampal ROIs were both located in the posterior hippocampus in the body. In the right hemisphere, activity in this region of the hippocampus has been shown to be associated with the accuracy of way finding performance (Hartley, Maguire, Spiers, & Burgess, 2003) and gray matter volume in this region of the hippocampus has been associated with time as a London taxi driver (Maguire, Woollett, & Spiers, 2006). In the left hemisphere, greater activity in this region of the hippocampus has been found during first person perspective navigation vs. survey perspective navigation (Sherrill et al., 2013). The right and left retrosplenial cortex ROIs were located in a region of the retrosplenial cortex just posterior to the splenium of the corpus callosum in the isthmus cingulate cortex and covered a region that did not extend into or posterior to the parieto‐occipital sulcus. In the left hemisphere, activity in this region of the retrosplenial cortex during a delay has been shown to be associated with the degree of maintenance of self‐rotation information (Chrastil et al., 2016). In both hemispheres, greater activity in this region of the retrosplenial cortex has been found during first person perspective navigation vs. survey perspective navigation, as well (Sherrill et al., 2013).

To create the brain activation map used to define these ROIs, the preprocessed data were prewhitened and a double‐gamma HRF convolution was applied to the stimulus waveform representing VPI in each run. The temporal derivative was included in the design matrix. The regressors for VPI did not include the 6‐s response period. The response period was grouped with the fixation cross period, and thus, only the active VPI periods were modeled. FSL's FLAME 1 + 2 with a Z threshold of 2.3 and a FWER‐corrected cluster p threshold of 0.05 was used in the group‐level analysis. Prethreshold masking was carried out using a gray matter mask created from the probabilistic gray matter tissue prior included in FSL v5.0.8. The gray matter mask was created by thresholding the gray matter tissue prior to only include voxels with an intensity equal to 100 or greater. Voxel intensity in this prior map reflects the likelihood that a voxel is gray matter and ranges from approximately 1 to 240.

We predicted that functional connectivity strength between our OF‐sensitive regions and primary auditory cortex during VPI would not be associated with navigation ability and selected these regions as control ROIs. We selected the center coordinates of these ROIs in the left and right hemispheres using the Harvard‐Oxford Cortical Structural Atlas included in FSLview. Specifically, these coordinates were located in left and right Heschl's gyri and are listed in Table 1. Binary spheres with a radius of 5 mm were centered at these coordinates and are shown in Figure 2.

#### 2.5.4 Psychophysiological interactions analyses

Psychophysiological interactions (PPI) analyses were performed to calculate task‐related functional connectivity strength between OF‐sensitive ROIs and our target and control ROIs (O'Reilly et al., 2012). PPI analyses were performed for VPI and TC runs using each OF‐sensitive region (L pV6, R pV6, L CSv, R CSv, R Pc, R pVIP) as a seed region in order to generate measures of functional connectivity strength between each OF‐sensitive region and each target and control ROI during each task. The functional connectivity measures extracted from these PPI analyses represent functional connectivity during either VPI or TC relative to rest (white fixation cross on a black background). The PPI analyses were run in FSL as follows. For our first level analyses, a set of explanatory variables (EVs) was generated for each OF‐sensitive seed for each run. The first EV (EV1) modeled VPI or TC task blocks. EV1 did not include the 6‐s response period (i.e., the response period was grouped with the fixation cross period). Only the active VPI and TC periods were modeled, which resulted in the PPI maps reflecting functional connectivity strength between regions while participants viewed each path and either path integrated or counted turns. The temporal derivative of EV1 was included in the model, which improves the model fit. The second EV (EV2) for each PPI analysis consisted of the extracted time series from a given OF‐sensitive ROI (L pV6, R pV6, L CSv, R CSv, R Pc, R pVIP) in the image space of that particular participant and run. The third EV (EV3) modeled the interaction between EV1 and EV2 (i.e., the PPI). The preprocessed data were prewhitened, temporal filtering was performed, and a double‐gamma HRF convolution was applied to the stimulus waveform representing the task period in each run. Fixed‐effects analyses were performed for within‐subject, higher level analyses to obtain parameter estimate maps representing the functional connectivity strength between each OF‐sensitive region and the rest of the brain during VPI or TC. The average parameter estimate in each target and control ROI was extracted from each OF‐sensitive region functional connectivity map in each participant. These values were used as measures of functional connectivity strength between each OF‐sensitive ROI and each target and control ROI during VPI or TC relative to rest and were the measures used to test our hypothesis.

#### 2.5.5 Relationship between SBSoD score and functional connectivity strength

The relationship between self‐reported spatial navigation ability and functional connectivity strength during VPI and TC was examined in two ways. First, we tested our hypothesis by directly examining the relationship between SBSoD score and functional connectivity strength between each OF‐sensitive region and our target and control ROIs during the VPI task. Linear correlations were run between SBSoD score and functional connectivity strength between each OF‐sensitive region (L pV6, R pV6, L CSv, R CSv, R Pc, R pVIP) and each target ROI (left and right retrosplenial cortex and hippocampus) (24 correlations total) during VPI in MATLAB R2017a. One‐tailed tests were used due to our prediction that positive relationships between self‐reported navigation ability and task‐related functional connectivity strength between these regions would be found during VPI. The *p*‐values from significant tests are presented along with false discovery rate (FDR)‐adjusted *p*‐values (Yekutieli & Benjamini, 1999). The specificity of relationships significant at a *p* < 0.05 level to the VPI task (i.e., a task with navigational demands) was tested next. For only the significant relationships (3/24), the functional connectivity strength between those OF‐sensitive ROIs and target ROIs during TC was extracted and linear correlations were run between these measures and SBSoD score. One‐tailed tests were performed for the sake of direct comparison to the results found for the VPI task. The *R* ^2^ and *p*‐values of these relationships were compared between VPI and TC. Lastly, linear correlations were run between SBSoD score and functional connectivity strength between each OF‐sensitive region and each control ROI (left and right primary auditory cortex) (12 correlations) during VPI as control analyses.

The second way we examined the relationship between self‐reported spatial navigation ability and OF‐sensitive region functional connectivity strength was through exploratory whole‐brain analyses. These analyses were performed to examine whether the FC strength between any OF‐sensitive regions and other brain regions beyond our target ROIs were positively or inversely associated with self‐reported spatial navigation ability during VPI or TC. In the context of VPI, these analyses reveal brain regions whose interactions with OF‐sensitive regions are stronger or weaker in better self‐reported navigators while using visual information to path integrate. In the context of TC, these analyses reveal similar relationships, but reflect interactions between brain regions while participants viewed stimuli compatible with egomotion while not having to use the information to track their location. Very little is known about how OF‐sensitive region functional connectivity patterns might differ when egomotion‐compatible stimuli are used to achieve different goals. Even less is known about how brains may achieve this differently. Our goal in these analyses was to explore these possibilities. Participants’ VPI and TC PPI maps for each OF‐sensitive region (six for VPI, six for TC) were entered into a group‐level analysis in which the positive and inverse effects of SBSoD score on these maps were examined. FSL's FLAME 1 + 2 was used to carry out these analyses with a Z threshold of 2.3 and a FWER‐corrected cluster p threshold of 0.05, and these are the main results reported due to the exploratory nature of these analyses. These analyses were also run with a more stringent Z threshold of 3.1 and a FWER‐corrected cluster p threshold of 0.05, and these results are summarized. Prethreshold masking was carried out using the gray matter mask discussed under Defining Target and Control ROIs. This set of exploratory results relies on cluster thresholding. In FSL, Gaussian Random Field Theory is used with a statistical map (of Z values), a cluster‐defining threshold (Z threshold), and an estimate of image smoothness to determine the FWER‐corrected *p*‐value for each cluster (Jezzard, Matthews, & Smith, 2001).

As part of the visualization of the results of these exploratory analyses, we created summary maps to provide an overview of where functional connectivity strength of OF‐sensitive regions has a significant positive or inverse association with self‐reported spatial navigation ability. To create these summary maps, the group‐level VPI > rest and TC > rest SBSoD effect maps (described in the previous paragraph) showing significant results were binarized and summed. Thus, in these summary maps, the number within each voxel represents the number of OF‐sensitive regions (i.e., 0–6) whose functional connectivity strength with that voxel was associated with SBSoD score during VPI or TC.

## 3 RESULTS

### 3.1 Task performance and strategy

Performance on the VPI and TC tasks during practice runs and in the scanner was highly accurate, in line with our intentions in designing this task. The mean per cent correct for VPI trials in the scanner was 95.4% (*SD* = 8.59%) and the mean percent correct for TC trials in the scanner was 91.3% (*SD* = 16.3%). The mean SBSoD score for this sample was 4.71 (*SD* = 1.17, range = 2.33–6.33). Neither SBSoD score (SBSoD_male_ = 4.68, SBSoD_female_ = 4.73, *p* = 0.941) nor performance on the VPI task (VPI_male_ = 98.1%, VPI_female_ = 92.4%, *p* = 0.270) or TC task (TC_male_ = 97.7%, TC_female_ = 83.9%, *p* = 0.154) was significantly different between males and females. Overall, 11/15 participants clearly reported using a homing strategy for the VPI task in which they described continually updating their representation of the starting location throughout each trial. This suggests that they maintained an internal representation of the goal that was updated throughout the path. Of the four participants that did not clearly report such a strategy, two reported a visual imagery‐based strategy that involved envisioning the start location. The other two participants reported generating a mental image of the path and determining the straight line back to the starting location based on that image. Importantly, these four participants all reported visuospatial strategies that involved creating a “mental image” of the path or the starting location. Only one participant reported occasionally using street signs or parked cars on some trials but primarily used a homing strategy. Lastly, 9/15 participants reported that the neighborhood in the task was familiar to them when asked (i.e., they did not spontaneously report this as something that factored into their task strategy). Unprompted, one participant specifically reported that this familiarity did not help in performing the task.

### 3.2 Relationship between self‐reported spatial navigation ability and task‐related functional connectivity strength between OF‐sensitive regions and target ROIs

**Figure 3:**
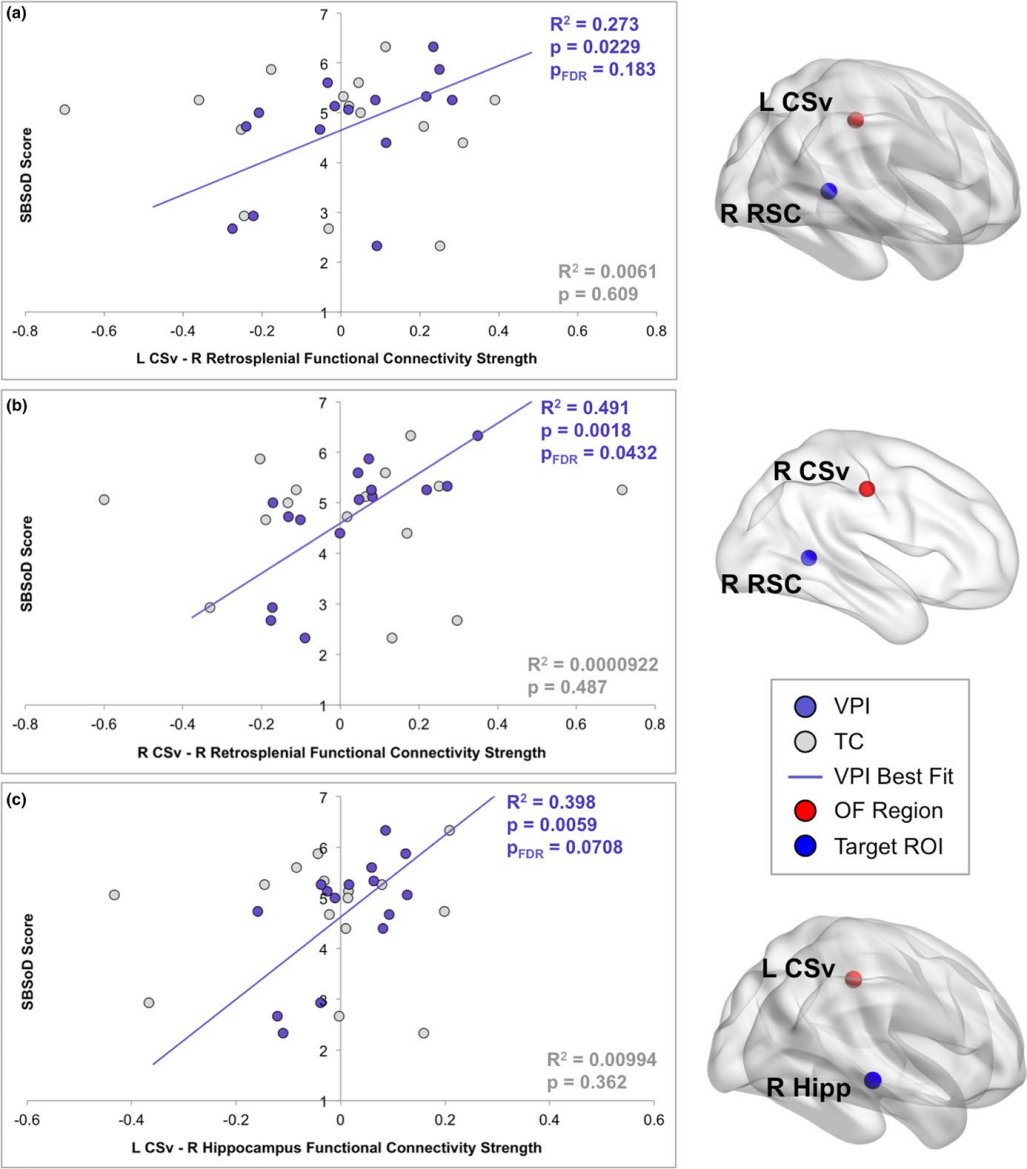
Self‐reported spatial navigation ability is associated with functional connectivity strength between L CSv and R CSv and navigationally relevant target regions during visual path integration. Pearson's correlations were run between SBSoD score and functional connectivity strength between each OF‐sensitive seed region and each target/control region during VPI. Three significant relationships were found and are shown in the figure; all involve the OF‐sensitive regions L CSv and R CSv. OF‐sensitive (red) and target (blue) ROIs whose interaction strength during VPI was positively correlated with SBSoD score are shown on cortical surfaces (right) created in BrainNet Viewer (Xia et al., 2013). Graphs a–c (left) plot the relationship between SBSoD score and task‐related functional connectivity strength between each pair of regions during VPI (purple) and TC (gray). The horizontal axes show the average parameter estimate of the PPI regressor for each functional connectivity analysis with positive values representing stronger functional connectivity between each OF‐sensitive seed region and each target region during VPI or TC relative to rest. The vertical axes show SBSoD scale score with larger scores representing better self‐reported spatial navigation ability. The *R* ^2^ values and *p‐*values of the relationships between SBSoD score and functional connectivity strength between each pair of regions during VPI are shown in purple alongside the line of best fit (purple). The *R* ^2^ values and *p*‐values of the relationships between SBSoD score and functional connectivity strength between each pair of regions during TC are shown in gray in the lower right hand corner of each graph. FDR: false discovery rate; L CSv: left cingulate sulcus visual area; OF: optic flow; PPI: psychophysiological interaction; R CSv: right cingulate sulcus visual area; R Hipp: right hippocampus; ROI: region of interest; R RSC: right retrosplenial cortex; SBSoD: Santa Barbara Sense of Direction Scale; TC: turn counting; VPI: visual path integration

The relationships between SBSoD score and functional connectivity strength between three OF‐sensitive regions and target regions during VPI were significant at *p* < 0.05 (L CSv − R retrosplenial: *p* = 0.0229; R CSv − R retrosplenial: *p* = 0.0018; L CSv − R hippocampus: *p* = 0.0059; 3/24 hypothesized relationships). The relationship between SBSoD score and functional connectivity strength between R CSv and right hippocampus trended toward significance with *p* = 0.0869. After calculating FDR‐adjusted *p*‐values amongst hypothesized relationships, one relationship remained significant (R CSv − R retrosplenial, *p*_FDR_ = 0.0432) and one trended toward significance (L CSv − R hippocampus, *p*_FDR_ = 0.0708). When the relationship between SBSoD score and functional connectivity strength between these regions during TC was examined, no relationship was significant or trended toward significance (*p *= 0.302–0.609). The significant relationships between SBSoD score and functional connectivity strength between L/R CSv and target ROIs during VPI and TC are plotted with lines of best fit (for VPI), *R* ^2^ values, and *p*‐values in Figure 3. When examining the relationship between SBSoD score and functional connectivity strength between OF‐sensitive regions and control ROIs in left and right primary auditory cortex during VPI, no *r* exceeded 0.116 and no relationships were significant, as expected.

### 3.3 Whole‐brain analyses: Relationship between self‐reported spatial navigation ability and OF‐sensitive region task‐related functional connectivity strength to other brain regions

**Figure 4:**
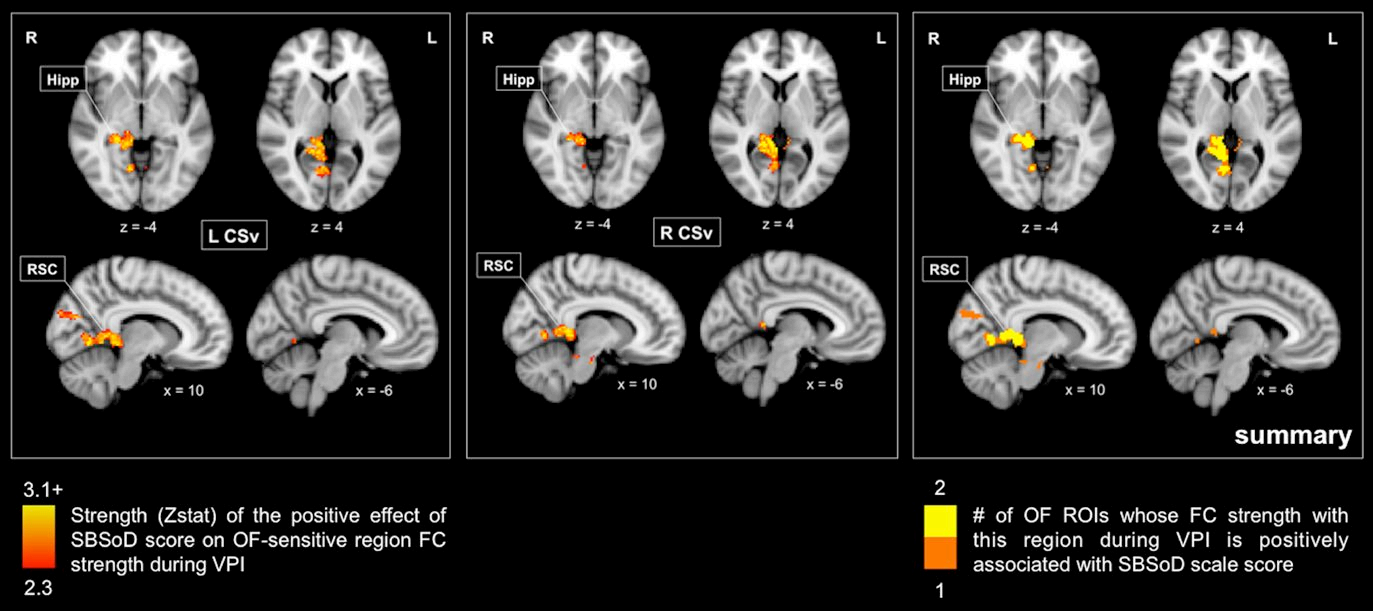
Whole‐brain analyses show positive relationships between self‐reported navigation ability and optic flow‐sensitive region functional connectivity strength during visual path integration. Effect maps (left and center) and summary map (right) showing where the functional connectivity patterns of OF‐sensitive regions during VPI were positively associated with SBSoD score (Z threshold 2.3). (left and center) Stronger connectivity between OF‐sensitive regions (L CSv and R CSv) and warm‐colored areas during VPI relative to rest was associated with better self‐reported navigation ability (higher SBSoD scores). (right) The maximum overlap of where OF‐sensitive region functional connectivity strength was positively associated with SBSoD score was 2, and this can be seen in right retrosplenial cortex, hippocampus, and lingual gyrus. Maps are displayed on the MNI152 T1 2 mm template, and x, y, and z slices correspond to this template. For more detailed information on the strength and statistical significance of the relationships shown in these figures, see Table 2. FC: functional connectivity; Hipp: hippocampus; L: left; L CSv: left cingulate sulcus visual area; MNI: Montreal Neurological Institute; OF: optic flow; R: right; R CSv: right cingulate sulcus visual area; ROI: region of interest; RSC: retrosplenial cortex; SBSoD: Santa Barbara Sense of Direction; VPI: visual path integration

Significant positive relationships between SBSoD score and L CSv and R CSv functional connectivity patterns during VPI were found and are shown in Figure 4. FWER‐corrected *p*‐values of significant clusters and peak *t* values within them are reported in Table 2. The functional connectivity strength between L CSv and right posterior hippocampus, right retrosplenial cortex, right lingual gyrus, and right cuneal cortex was positively associated with SBSoD score, which largely reflects the results of our ROI‐based analyses. Similarly, the functional connectivity strength between R CSv and right posterior hippocampus, right retrosplenial cortex, and right lingual gyrus was positively associated with SBSoD score. Importantly, no significant inverse relationships were found between SBSoD score and functional connectivity strength between any OF‐sensitive region and any other brain region during VPI. At the more stringent Z threshold of 3.1 and FWER‐corrected cluster p threshold of 0.05, functional connectivity strength between L CSv and right retrosplenial cortex and lingual gyrus was positively associated with SBSoD score. Similarly, functional connectivity strength between R CSv and right retrosplenial cortex was positively associated with SBSoD score.

**Table 2:** Associations between self‐reported navigation ability and functional connectivity patterns of optic flow‐sensitive regions during visual path integration

OF‐sensitive ROI	Region	Association with SBSoD score	MNI 152 coordinates (*x*, *y*, *z*)	*t*	Cluster (FWER‐corrected *p*‐value)
L CSv	Right hippocampus	Positive	26, −34, −4	3.72	1 (0.00424)
	Right retrosplenial cortex		10, −40, 2	3.63	1
	Right lingual gyrus		10, −52, 2	3.51	1
	Right cuneal cortex		8, −76, 22	3.04	2 (0.0477)
	Right occipital pole		20, −88, 30	3.47	2
R CSv	Right hippocampus	Positive	24, −34, −4	3.31	1 (0.00285)
	Right retrosplenial cortex		8, −44, 4	4.16	1
	Right lingual gyrus		6, −64, 4	3.57	1

Note

The significant effects of SBSoD score on OF‐sensitive region FC patterns during VPI are shown as well as the direction of the association (positive vs. inverse). The MNI152 coordinates of peak effects are shown alongside the t‐values of those peaks and the significance of the clusters (Z threshold 2.3) in which those peaks are located. Figure 4 shows individual and summary maps of these results.

FC: functional connectivity; FWER: family‐wise error rate; L CSv: left cingulate sulcus visual area; MNI: Montreal Neurological Institute; OF: optic flow; R CSv: right cingulate sulcus visual area; SBSoD: Santa Barbara Sense of Direction; VPI: visual path integration.

**Figure 5:**
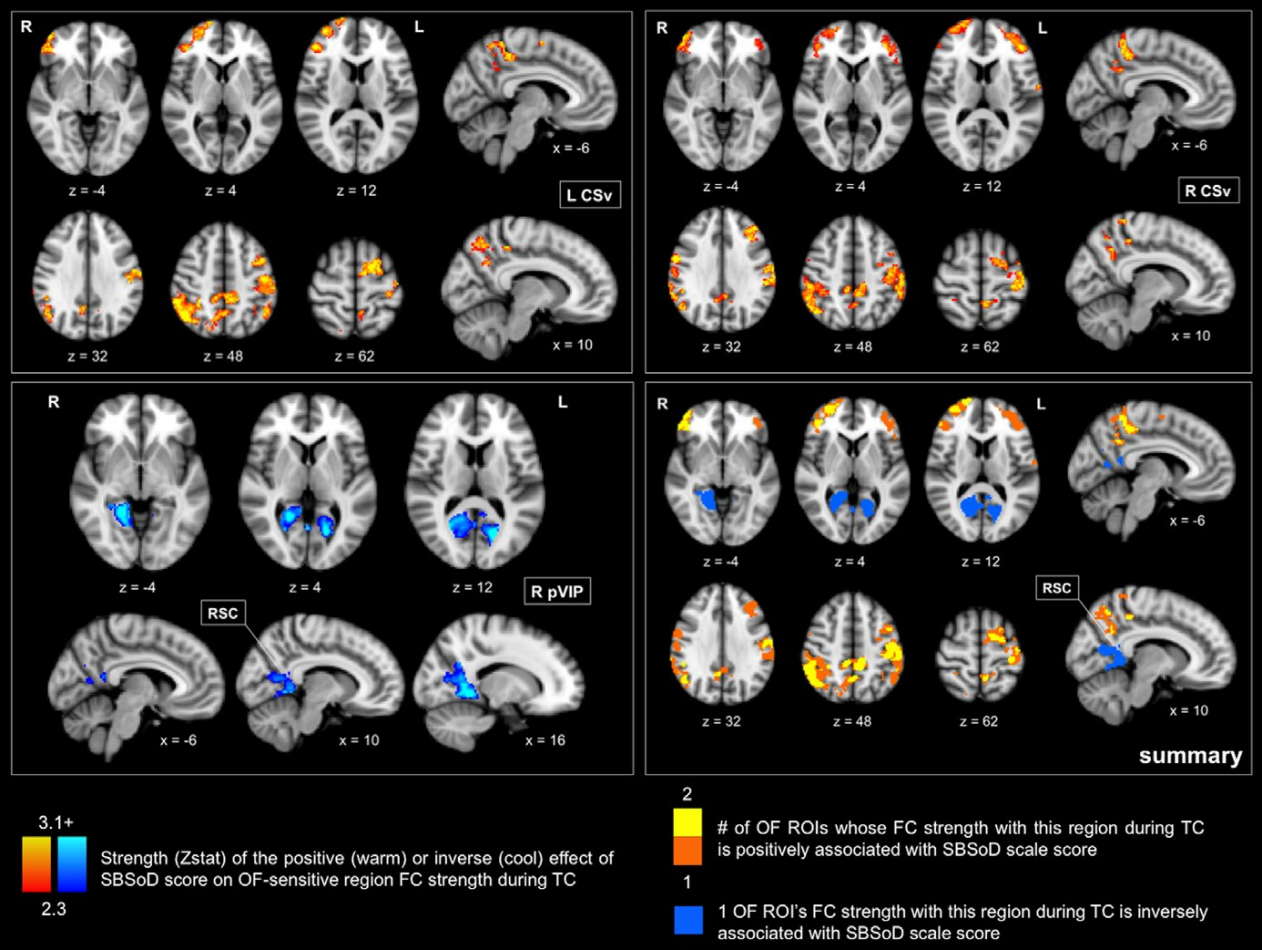
Whole‐brain analyses show positive and inverse relationships between self‐reported navigation ability and optic flow‐sensitive region functional connectivity strength during turn counting. Effect maps (left and top right) and summary map (bottom right) showing where the functional connectivity patterns of OF‐sensitive regions during TC were positively and inversely associated with SBSoD score (Z threshold 2.3). Warm colors represent a positive association; (top) stronger connectivity between OF‐sensitive regions (L CSv, R CSv) and warm‐colored areas during TC relative to rest was associated with better self‐reported navigation ability (higher SBSoD scores). (bottom right) The maximum overlap of where OF‐sensitive region functional connectivity was positively associated with SBSoD score was 2, which can be seen in a number of areas in the summary map. Blue regions represent the inverse association found; (bottom) stronger connectivity between the OF‐sensitive region R pVIP and cool‐colored areas during TC relative to rest was associated with worse self‐reported navigation ability (lower SBSoD scores). Maps are displayed on the MNI152 T1 2 mm template, and x, y, and z slices correspond to this template. For more detailed information on the strength and statistical significance of the relationships shown in these figures, see Table 3. FC: functional connectivity; Hipp: hippocampus; L: left; L CSv: left cingulate sulcus visual area; MNI: Montreal Neurological Institute; OF: optic flow; R: right; R CSv: right cingulate sulcus visual area; ROI: region of interest; RSC: retrosplenial cortex; SBSoD: Santa Barbara Sense of Direction; TC: turn counting

In contrast, both positive and inverse associations were found between SBSoD score and OF‐sensitive region functional connectivity strength with other brain areas during TC. These are shown in Figure 5, and FWER‐corrected *p*‐values of significant clusters and peak *t* values within them are reported in Table 3. There was a positive relationship between SBSoD score and functional connectivity strength between L CSv, R CSv, and other brain regions during TC, and there was an inverse relationship between SBSoD score and functional connectivity strength between R pVIP and other brain regions during TC. As shown in Figure 5 in warm colors, significant positive relationships were found between SBSoD score and functional connectivity strength between L CSv and regions in the lateral parietal lobe, precuneus, and lateral prefrontal cortex during TC. Similar relationships were found for R CSv. Shown in Figure 5 in blue, a significant inverse relationship was found between SBSoD score and functional connectivity strength between R pVIP and intracalcarine cortex, lingual gyrus, and retrosplenial cortex during TC. At the more stringent Z threshold of 3.1 and FWER‐corrected cluster p threshold of 0.05, functional connectivity strength between L CSv and right frontal pole, right lateral parietal cortex, and left precentral/postcentral gyri was positively associated with SBSoD score. At the more stringent threshold, functional connectivity strength between R CSv and left middle frontal gyrus and right lateral parietal cortex was positively associated with SBSoD score. Lastly, at the more stringent threshold, functional connectivity strength between R pVIP and right lingual gyrus and right retrosplenial cortex was inversely associated with SBSoD score.

**Table 3:** Associations between self‐reported navigation ability and functional connectivity patterns of optic flow‐sensitive regions during turn counting

OF‐sensitive ROI	Region	Association with SBSoD score	MNI 152 coordinates (*x*, *y*, *z*)	*t*	Cluster (FWER‐corrected *p*‐value)
L CSv	Right frontal pole	Positive	44, 40, 0	3.84	1 (0.00401)
			48, 38, 12	4.34	1
			36, 56, 12	4.22	1
			20, 66, 10	3.55	1
	Left postcentral gyrus		−46, −18, 38	4.15	2 (1.13 × 10^−5^)
			−48, −26, 54	3.83	2
	Left precentral gyrus		−46, −14, 46	5.04	2
			−38, −4, 54	4.63	2
	Left angular gyrus		−50, −52, 46	4.38	2
	Left middle frontal gyrus		−42, 6, 52	4.07	2
	Left superior frontal gyrus		−18, 0, 66	4.59	2
	Right angular gyrus		52, −58, 36	4.83	3 (1.79 × 10^−6^)
	Right supramarginal gyrus		50, −44, 40	4.78	3
			56, −42, 48	4.92	3
			50, −34, 54	4.65	3
	Right precuneus		4, −58, 50	3.91	3
			6, −56, 40	3.53	3
	Left precuneus		−8, −40, 46	3.96	3
R CSv	Right frontal pole		44, 52, −4	3.51	1 (0.00279)
			42, 40, 4	3.34	1
			34, 58, 10	3.99	1
			48, 40, 16	3.56	1
			34, 58, 16	3.97	1
	Left frontal orbital cortex		−28, 36, −12	3.77	2 (0.000213)
	Left frontal pole		−42, 38, 10	4.04	2
	Left middle frontal gyrus		−36, 32, 28	4.13	2
			−36, 26, 34	4.68	2
	Left postcentral gyrus		−64, −14, 30	4.12	3 (2.38 × 10^−6^)
			−44, −16, 46	3.87	3
			−34, −28, 62	3.89	3
	Left supramarginal gyrus		−60, −30, 36	3.71	3
			−54, −36, 52	3.85	3
	Left precentral gyrus		−32, −8, 52	4.37	3
			−42, −16, 64	3.68	3
	Left superior frontal gyrus		−16, 0, 64	3.92	3
	Right precentral gyrus		60, −2, 32	4.59	4 (9.89 × 10^−6^)
	Right postcentral gyrus		60, −16, 34	3.57	4
	Right angular gyrus		62, −50, 32	3.71	4
			48, −54, 40	4.19	4
	Right superior lateral occipital cortex		52, −62, 36	3.98	4
			46, −60, 50	3.78	4
	Right supramarginal gyrus		52, −44, 40	4.49	4
			58, −40, 44	4.40	4
	Right superior parietal lobule		28, −40, 54	3.58	4
	Right precuneus		8, −54, 32	3.51	5 (0.00452)
			8, −54, 38	3.55	5
			6, −58, 50	3.64	5
			6, −36, 50	3.48	5
	Right posterior cingulate gyrus		2, −46, 38	3.55	5
	Left posterior cingulate gyrus		−4, −50, 34	3.44	5
	Left precuneus		−6, −42, 50	4.27	5
R pVIP	Right lingual gyrus	Inverse	20, −46, −2	6.30	1 (2.18 × 10^−5^)
	Right precuneus		20, −54, 6	4.44	1
			18, −54, 16	3.55	1
	Right retrosplenial cortex		10, −46, 2	3.56	1
	Left intracalcarine cortex		−18, −68, 8	6.59	1
	Left retrosplenial cortex		−6, −50, 14	3.42	1

Note

The significant effects of SBSoD score on OF‐sensitive region FC patterns during TC are shown as well as the direction of the association (positive vs. inverse). The MNI152 coordinates of peak effects are shown alongside the *t *values of those peaks and the significance of the clusters (Z threshold 2.3) in which those peaks are located. Figure 5 shows individual and summary maps of these results.

FC: functional connectivity; FWER: family‐wise error rate; L CSv: left cingulate sulcus visual area; MNI: Montreal Neurological Institute; OF: optic flow; R CSv: right cingulate sulcus visual area; R pVIP: right putative ventral intraparietal area; SBSoD: Santa Barbara Sense of Direction; TC: turn counting.

## 4 DISCUSSION

Here, we present novel evidence that perceived spatial navigation ability is sensitive to functional connectivity strength between OF‐sensitive brain regions and navigationally relevant brain regions during visual path integration. Our ROI‐based analyses showed a positive association between self‐reported spatial navigation ability and functional connectivity strength between L/R CSv and right retrosplenial cortex and posterior hippocampus during a task with navigational demands. These relationships were specific to our VPI task. These results support our hypothesis that better self‐reported spatial navigation ability is associated with stronger communication between OF‐sensitive regions and regions important to navigation during visual path integration. Our exploratory results revealed similar positive relationships between self‐reported navigation ability and OF‐sensitive region functional connectivity strength during VPI. On the other hand, during TC, our exploratory results showed both positive and inverse relationships between self‐reported spatial navigation ability and OF‐sensitive region functional connectivity strength. These results suggest that functional connectivity patterns of OF‐sensitive regions that are specifically sensitive to egomotion‐compatible stimuli vary according to self‐reported navigation ability while viewing navigationally relevant stimuli even in the absence of navigational demands.

### 4.1 Self‐reported navigation ability is positively associated with functional connectivity strength between L CSv and R CSv and right retrosplenial cortex and posterior hippocampus during VPI

Self‐reported navigation ability was associated with communication strength between L/R CSv and right retrosplenial cortex and L CSv and right posterior hippocampus selectively during VPI—suggesting that information transfer between these regions during large‐scale spatial tasks is at least partially associated with perceived navigation ability. Additionally, the relationship between SBSoD score and functional connectivity strength between R CSv and right posterior hippocampus trended toward significance. While the precise aspects of visual motion processing that occur in L/R CSv are not known, it is thought that these regions are important to using visual information to process self‐motion. L/R CSv are highly selective for egomotion‐compatible stimuli in that they do not respond to egomotion‐incompatible stimuli (Cardin & Smith, 2009) and are inhibited by random motion (Pitzalis et al., 2013). We were able to define L/R CSv because these regions responded more strongly to coherent dot motion (consistent with egomotion) than scrambled dot motion (inconsistent with egomotion), which is consistent with the egomotion selectivity of these areas. Figure S1 additionally shows that L CSv and R CSv overlapped with regions that responded more strongly during VPI (which contains egomotion) than during a still counting task (which did not contain any motion) in an independent sample of five participants. In this still counting task, participants were presented with series of still images taken from videos similar to those shown in VPI and were asked to count the number of still images presented in each series. L CSv and R CSv also overlapped with regions that responded more strongly during TC (which contains the same egomotion present in VPI) compared to the still counting task. This provides confidence that L CSv and R CSv were responding to the visual egomotion present in both VPI and TC tasks. Though our experiment did not directly assess this, it is likely that information flows from L/R CSv, in which aspects of egomotion are processed, to right retrosplenial cortex and posterior hippocampus, which use this information to generate a representation of space and/or one's position within it. Through this lens, stronger functional connectivity between L/R CSv and right retrosplenial cortex and posterior hippocampus during VPI in self‐reported better navigators may reflect better translation of visual self‐motion information into spatial representations.

Our hypothesis‐driven analyses were focused on the retrosplenial cortex and hippocampus as target regions because numerous neuroimaging and lesion studies support the role of the retrosplenial cortex and hippocampus in spatial navigation. Retrosplenial cortex is thought to play a role in understanding one's position within an environment, specifically connecting spatial context and directional information with visual cues, coding location and facing direction within environments, and forming and using cognitive maps (Epstein, 2008; Epstein et al., 2017; Iaria, Chen, Guariglia, Ptito, & Petrides, 2007; Ino et al., 2007; Marchette, Vass, Ryan, & Epstein, 2014; Takahashi, Kawamura, Shiota, Kasahata, & Hirayama, 1997; Vann, Aggleton, & Maguire, 2009; Wolbers & Buchel, 2005). Increased retrosplenial cortex activity during environmental spatial tasks is associated with better performance on those tasks (Auger, Zeidman, & Maguire, 2017; Moffat, Elkins, & Resnick, 2006). Retrosplenial cortex activity is also associated with the amount of information learned about an environment experienced in first person perspective (Wolbers & Buchel, 2005). Retrosplenial cortex has recently been proposed to be an important hub in networks supporting spatial navigation processes (Ekstrom et al., 2017), and preliminary evidence supporting this exists (Kong et al., 2017). Furthermore, head direction cells have been found in rodent retrosplenial cortex (Chen, Lin, Green, Barnes, & McNaughton, 1994; Cho & Sharp, 2001), supporting its role in understanding one's position within the environment. While both left and right retrosplenial cortex have been shown to support spatial navigation skills, right retrosplenial cortex may be particularly important (see Maguire, 2001 for review). Individuals who develop right retrosplenial cortex lesions are likely to develop topographical disorientation—though this tends to resolve if left retrosplenial cortex is still intact (Maguire, 2001; Takahashi et al., 1997). The selective association between perceived navigation ability and interaction strength between L/R CSv and right retrosplenial cortex during VPI is congruent with the role of right retrosplenial cortex in understanding one's orientation within an environment.

The hippocampus has long been implicated in spatial navigation and is thought to be a region in which cognitive maps are formed due to the existence of place cells within rodent (O'Keefe & Dostrovsky, 1971), nonhuman primate (Ludvig, Tang, Gohil, & Botero, 2004; Matsumura et al., 1999), and human hippocampi (Ekstrom et al., 2003). Similar to retrosplenial cortex, both hippocampi have been shown to be involved in performing spatial navigation tasks, though the right hippocampus has been implicated as selectively important in some contexts. London taxi drivers, who are required to form and frequently use highly detailed cognitive maps of London, have higher gray matter density in right posterior hippocampus compared to bus drivers and controls (Maguire et al., 2000, 2006). Iaria et al. (2007) found right posterior hippocampus to be specifically involved in using a cognitive map to navigate within a virtual environment. In another study, participants who underwent right temporal lobectomies showed topological memory impairment while those who underwent left temporal lobectomies showed episodic memory impairment (Spiers et al., 2001). Specifically related to this study, individuals who underwent right hippocampal lobectomies performed worse on path integration tasks compared to those with intact hippocampi and those who underwent left hippocampal lobectomies (Philbeck et al., 2004; Worsley et al., 2001). The association between perceived navigation ability and interaction strength between L CSv and right posterior hippocampus selectively during VPI aligns with the role of right hippocampus in navigation‐based processes.

Additional evidence for the importance of right retrosplenial cortex and hippocampus to spatial navigation ability has been found through studying the brain at rest. One recent fMRI study showed that better self‐reported navigators had stronger functional interactions between right posterior hippocampus and right retrosplenial cortex (but not left) at rest (Sulpizio et al., 2016). Another resting‐state fMRI study found right retrosplenial cortex to be the largest hub within a navigation brain network and that the betweenness centrality of right retrosplenial cortex in this navigation network was higher in better self‐reported navigators (Kong et al., 2017). Betweenness centrality of a brain region is a measure of network configuration that reflects the fraction of shortest paths between regions in the network that contain the brain region of interest (Rubinov & Sporns, 2010), and this is thought to reflect information transfer among nodes in a given network. Thus, this result suggests that perceived navigation ability is associated with the resting interconnectivity of right retrosplenial cortex within a network containing brain regions involved in spatial navigation. It is possible that the configuration of right retrosplenial cortex within this brain network at rest is associated with its interactions with other brain regions during navigation tasks, and this should be examined in future work. Complementary to these resting‐state studies, our results support that stronger task‐related interactions between right retrosplenial cortex and posterior hippocampus and specific visual motion areas are associated with better self‐reported navigation ability.

The retrosplenial cortex and hippocampus are thought to support survey, or allocentric, representations of space, which are viewpoint‐independent, flexible, and tend to be used by individuals who navigate space well (Galati et al., 2000; Hartley et al., 2003; Iaria et al., 2007; Maguire et al., 1998; Marchette, Bakker, & Shelton, 2011; Ohnishi, Matsuda, Hirakata, & Ugawa, 2006; Wolbers & Buchel, 2005). It is possible that self‐reported better navigators automatically engage systems that are tuned to support allocentric representations of large‐scale space in navigational contexts. In support of this, Arnold et al. (2013) found a significant positive correlation between SBSoD score and accuracy of cognitive map formation, which is a typical measure of allocentric spatial representations. This suggests that self‐reported better navigators tend to form more accurate allocentric representations of space. In our study, self‐reported navigation ability was associated with the communication strength between L/R CSv and regions that support allocentric spatial representations during VPI. This could mean that self‐reported better navigators automatically started to build allocentric spatial representations even though this was not necessary to accurately complete the VPI task. A consistent but slightly different interpretation is related to the fact that survey representations require metric information about the environment they represent (i.e., knowledge of the relative distances between locations/landmarks in an environment) (Chrastil, 2013). Stronger functional connectivity between L/R CSv and allocentric regions during VPI could reflect better extraction of environmental metric information from visual motion. They could also reflect greater reliance on L/R CSv for visual motion processing, which in turn may also reflect more accurate extraction of metric information from visual motion, which is important to the formation of accurate survey representations. More work is needed to better understand the processes supported by interactions between L/R CSv and right retrosplenial cortex and posterior hippocampus in the context of spatial navigation tasks.

### 4.2 Whole‐brain results: Relationship between self‐reported navigation ability and functional connectivity patterns of L CSv and R CSv during VPI

In addition to our hypothesis‐driven ROI‐based analyses, we also explored whether self‐reported spatial navigation ability was associated with functional connectivity strength between any OF‐sensitive regions and brain regions beyond our target ROIs. The results of our whole‐brain analyses were very similar to those from our ROI‐based analyses for VPI. Only positive associations were found between functional connectivity patterns of OF‐sensitive regions and self‐reported navigation ability (though we also tested for inverse relationships). Associations were found between self‐reported navigation ability and functional connectivity strength between L CSv and R CSv and right posterior hippocampus, right retrosplenial cortex, and additionally right lingual gyrus. The region of the right posterior hippocampus whose functional connectivity strength with both L CSv and R CSv during VPI is associated with self‐reported navigation ability corresponds to a region that has been shown to be sensitive to distance from a goal location (Sherrill et al., 2013). This result was specific to VPI, suggesting that communication between these regions represents a process that is specifically beneficial in navigational contexts. The lingual gyrus has been broadly associated with spatial navigation; however, the interpretation of the involvement of this region and the regions in the right cuneal and occipital pole in this set of results is not clear. Two meta‐analyses have reported that several fMRI studies of spatial navigation have shown the involvement the lingual gyrus in diverse tasks (Boccia, Nemmi, & Guariglia, 2014; Kong et al., 2017). Boccia et al. (2014) additionally showed that this region has been implicated in both egocentric and allocentric representations. Overall, these whole‐brain results are congruent with our ROI‐based analyses showing that stronger interactions between L/R CSv and right hemisphere regions that support allocentric spatial representations are present in better self‐reported navigators in navigational contexts.

### 4.3 Whole‐brain results: Relationship between self‐reported navigation ability and functional connectivity patterns of OF‐sensitive regions during TC

Our whole‐brain analyses revealed both positive and inverse relationships between self‐reported navigation ability and functional connectivity strength between OF‐sensitive regions and several brain regions during TC. This suggests the presence of efficient and inefficient network configurations for processing the experience of short paths in first person perspective in the absence of navigational demands. We interpret these findings to be associated with general egocentric processes that occur during TC.

Until this point, our discussion has focused on the OF‐sensitive regions L CSv and R CSv because the functional connectivity patterns of other OF‐sensitive regions were not found to be associated with self‐reported spatial navigation ability in our VPI‐focused analyses. In this whole‐brain analysis, we found positive relationships involving L/R CSv functional connectivity patterns as well as an inverse relationship involving the functional connectivity pattern of R pVIP during TC. In contrast to L/R CSv being highly selective for egomotion‐compatible stimuli, Cardin and Smith (2009) found that R pVIP responded strongly to egomotion‐compatible stimuli, but that it also responded to egomotion‐incompatible stimuli, suggesting that it may have a more general role in visual motion processing. Interestingly, the relationships between self‐reported navigation ability and the functional connectivity patterns of these three OF‐sensitive regions during TC reflect these selectivities for egomotion‐compatible stimuli. Positive relationships were found between self‐reported navigation ability and functional connectivity strength between the highly egomotion‐sensitive regions L CSv and R CSv and regions that are largely part of the frontoparietal and somatomotor networks (Yeo et al., 2011) during TC. An inverse relationship was found between self‐reported navigation ability and functional connectivity strength between the less egomotion‐sensitive region R pVIP and retrosplenial cortex as well as intracalcarine cortex and lingual gyrus during TC. Worse self‐reported navigators may rely more on the information derived from visual motion processes that occur in R pVIP, which *is not* highly selective for egomotion‐compatible stimuli, than L CSv or R CSv, which *are* highly selective for egomotion‐compatible stimuli. Similar to L CSv and R CSv, Figure S1 shows that R pVIP responded more strongly during VPI and TC than during a still counting task. Thus, it is likely that R pVIP was also involved in aspects of visual motion processing during the TC task.

The positive association between perceived navigation ability and functional connectivity strength between L/R CSv and right retrosplenial cortex and posterior hippocampus during VPI and between L/R CSv and bilateral posterior parietal cortex during TC resemble ideas embedded within a network model of spatial navigation recently proposed by Ekstrom et al. (2017). They proposed that during allocentric‐heavy processes, retrosplenial cortex becomes more hub‐like within the navigation brain network, while during egocentric‐heavy processes, posterior parietal cortex becomes more hub‐like within the same navigation network. While we did not directly assess this hypothesis, it emphasizes the importance of neural context. Interaction strength between L/R CSv and different regions (i.e., contexts) in VPI and TC was associated with perceived navigation ability. More specifically, there was a relationship between perceived spatial navigation ability and functional connectivity strength between L/R CSv and right retrosplenial cortex during VPI (which may involve allocentric representation to some extent) and a relationship between L/R CSv and bilateral posterior parietal cortex during TC (which we assume largely involves egocentric representations). The relationship between perceived navigation ability and L/R CSv interaction strength with allocentric‐associated and egocentric‐associated regions in VPI and TC, respectively, resembles Ekstrom and colleagues’ network‐based conceptualization of allocentric and egocentric processes in spatial navigation contexts. This supports the continued investigation of network dynamics in the study of human spatial navigation.

During TC, self‐reported navigation ability was positively associated with functional connectivity strength between L CSv, R CSv, and bilateral precuneus as well as bilateral lateral frontoparietal regions that mostly overlapped with frontoparietal and somatomotor brain networks. Functional connectivity strength between L/R CSv and bilateral precuneus and right lateral parietal cortex (specifically angular and supramarginal gyri) was positively associated with self‐reported navigation ability. These parietal areas have largely been associated with egocentric representations of space (Chadwick, Jolly, Amos, Hassabis, & Spiers, 2015; Galati et al., 2000; Maguire et al., 1998; Ohnishi et al., 2006; Schindler & Bartels, 2013; Wolbers, Hegarty, Büchel, & Loomis, 2008). The regions in the precuneus whose connectivity strength with L CSv or R CSv showed this relationship have been shown to be associated with successful navigation to a goal in first person perspective (Sherrill et al., 2013), egocentric coding of space and goal locations (Chadwick et al., 2015; Schindler & Bartels, 2013), accurate path integration (Chrastil et al., 2015), and distance tracking (Chrastil et al., 2015, 2016). Lateral frontal regions have also been associated with egocentric representations, and there were positive relationships between self‐reported navigation ability and functional connectivity strength between L/R CSv and left lateral frontal regions that were in the vicinity of such egocentric coding regions (Schindler & Bartels, 2013). Interactions between L/R CSv and postcentral gyrus suggest that they may support the incorporation of somatosensory, or idiothetic, self‐motion cues into egocentric spatial representations when these cues are present. Taken together, these results suggest that while viewing paths in first person perspective without navigational demands, perceived navigation ability is associated with the communication strength between egomotion‐sensitive regions (L CSv, R CSv) and regions that contribute to egocentric representations of space. Stronger interactions between these regions may reflect more accurate conversion of visual self‐motion information into spatial representations.

An inverse relationship was present between self‐reported spatial navigation ability and functional connectivity strength between R pVIP and retrosplenial cortex, among other regions, during TC. In other words, stronger communication between R pVIP and retrosplenial cortex during TC was associated with worse self‐perceived navigation ability. As discussed in the context of our ROI‐based results, retrosplenial cortex is important to spatial navigation, associated with allocentric spatial representations, and its recruitment during navigation tasks is usually associated with better navigation ability and/or task performance. Thus, it is surprising that the interaction strength between R pVIP and retrosplenial cortex during TC was inversely related to self‐reported navigation ability. Broadly, these results support a neural context hypothesis of brain function (McIntosh, 2004) in that the interactions between right retrosplenial cortex and OF‐sensitive brain regions may be efficient or inefficient while viewing navigationally relevant stimuli depending on the navigational demands present (i.e., the context). In other words, increased task‐related interactions between right retrosplenial cortex and other brain regions are not always beneficial, even though right retrosplenial cortex is important to navigation ability and greater betweenness centrality of this region at rest is associated with better self‐reported navigation ability (Kong et al., 2017). One interpretation of this finding is that worse self‐reported navigators may rely more on R pVIP for processing visual motion when viewing egomotion‐compatible stimuli, and the computations that occur in R pVIP may produce less accurate estimations of self‐motion from visual motion than other regions. This hypothetical reliance on R pVIP for processing egomotion may lead to stronger connections (direct or indirect) between R pVIP and retrosplenial cortex in these individuals, though this is only speculation. In turn, it may be more difficult to down‐regulate interactions between these regions in the wrong context. More research into the computations performed in these OF‐sensitive regions and their interactions with the rest of the brain during different navigational tasks is needed to understand these relationships in greater detail.

### 4.4 Limitations and caveats

There are some limitations that readers should keep in mind as they consider our results. First, the order of VPI and TC tasks was not counterbalanced across participants in this study. In order to minimize the impact this could have on the results, no direct comparisons between VPI and TC conditions were made, and we limited our analyses to the association between SBSoD scores and functional connectivity strength within each condition rather than between conditions. Despite this, it is still possible that the lack of counterbalancing could have systematically affected functional connectivity strength during TC in a way that was related to SBSoD score. Second, the sample size in this experiment was modest, though similar in size to other published fMRI studies on spatial navigation and cognition. Future studies should aim to replicate these findings in independent and larger samples. Third, participants were only presented with visual stimuli during our tasks, and our findings support the existence of different configurations of networks recruited during VPI and TC in self‐reported good and poor navigators. Yet, it is unclear what these networks would look like if proprioceptive, vestibular, or peripheral visual input were present or involved in the greater experimental design (Shine, Valdes‐Herrera, Hegarty, & Wolbers, 2016). Unfortunately, this is a current limitation of any fMRI experiment designed to study spatial navigation due to the restrictive environment found within the MRI scanner; however, it does provide the advantage of isolating sensory input to one source. Perhaps as other technologies mature, it will be possible to study this in a more realistic fashion. Fourth, OF‐sensitive regions were not defined at a participant‐specific level in this study; doing so would have significantly increased scan time and participant burden beyond an acceptable level. Now that at least two published studies (Sherrill et al., 2015 and the present study) have linked OF‐sensitive and navigationally responsive regions during navigation tasks in humans, it would be valuable to define OF‐sensitive regions at the individual level to test more refined hypotheses about them. Research that could help us gain additional knowledge of the computations that take place in these visual motion areas would also allow for more specific hypotheses to be developed, and in turn, more targeted analyses to be performed. In this study, we used the criterion of sensitivity to egomotion‐compatible stimuli to meaningfully narrow down the OF‐sensitive ROIs included in our analyses to six OF‐sensitive ROIs, yet this is still a large number. Interestingly, all of our ROI‐based results involved L CSv and R CSv and the majority of our exploratory whole‐brain analyses involved these regions, as well, lending confidence to the role of CSv in spatial navigation ability in certain contexts.

Another caveat is our use of the same data to functionally define our navigationally relevant target ROIs (based on increased brain activity during the VPI task) and to test our hypotheses related to functional connectivity involving these ROIs during the VPI task. Though activity and connectivity measure different entities, this decision could have introduced some bias into our results. However, a few points suggest that the concern for bias is minimal. The first is that our whole brain analyses examining the effect of SBSoD score on functional connectivity patterns of L CSv and R CSv support the results of our ROI‐based analyses related to right retrosplenial cortex and posterior hippocampus. The second is that the results are specific to the right hemisphere, which is supported by the spatial navigation literature, even though we used activity during the VPI task to define retrosplenial cortex and hippocampal ROIs in the left hemisphere, as well. The third is that SBSoD score was not significantly associated with activity in our right retrosplenial cortex or in our right hippocampal ROIs during VPI (*p*_one‐tailed_ > 0.12). This provides support that activity in these ROIs during VPI is not fully responsible for the relationships found between functional connectivity strength between these ROIs and L/R CSv during VPI and self‐reported navigation ability.

Lastly, where plausible, future work should include objective measures of spatial navigation ability in addition to subjective ones in order to determine whether relationships between these measures and task‐related functional connectivity patterns of OF‐sensitive regions are similar. While some have shown that self‐reported navigation ability does not correspond well to objective spatial task performance (Takeuchi, 1992; Thorndyke & Goldin, 1981), many others have shown that self‐reported navigation ability does correspond to objective performance on spatial tasks in a young population (Kozlowski & Bryant, 1977; Sholl, 1988; see Hegarty et al., 2002 for additional discussion of this). Furthermore, the SBSoD scale was developed with these discrepancies in mind and tested to verify that it corresponds to performance on real‐world orientation tasks (Hegarty et al., 2002). Despite this, we caution that our results should only be interpreted in the context of self‐perceived navigation ability. Though one's perceived navigation ability is presumably developed through real‐world experiences, other factors certainly play a role in self‐perception. Objective measures of spatial navigation ability would be valuable in assessing this.

Beyond these limitations, there are some benefits to using the SBSoD in this experiment. Because the SBSoD scale measures general “sense of direction,” it is less limiting than a more specific measure, such as cognitive map formation or triangle completion error, might be. In this way, the results reported herein are potentially more generalizable. Another benefit is that the SBSoD has been used in other fMRI studies of navigation and therefore our results can be easily compared to these studies. Others have examined SBSoD score in the context of navigation fMRI tasks (Auger et al., 2012; Epstein et al., 2005; Halko et al., 2014; Janzen, Jansen, & Turennout, 2008) as well as the relationship between resting‐state functional connectivity patterns and SBSoD score (Kong et al., 2017; Sulpizio et al., 2016; Wegman & Janzen, 2011). Thus, our novel report of a relationship between SBSoD score and OF‐sensitive region functional connectivity patterns during our tasks can be contextualized within existing fMRI literature documenting a relationship between SBSoD score and brain activity and connectivity.

## 5 CONCLUSIONS

Our results present novel findings on potential neural mechanisms to which self‐perceived navigation ability is sensitive. Our ROI‐based results support our hypothesis that the strength of communication between OF‐sensitive brain regions and navigationally relevant brain regions during visual path integration is positively associated with perceived spatial navigation ability due to better information transfer between these regions in navigational contexts. The relationships involving functional connectivity between L/R CSv and right retrosplenial cortex and posterior hippocampus were specific to VPI. We interpret these relationships as relating to more accurate transformation of self‐motion information extracted from visual cues into mental representations of space or distance traveled. Our VPI whole‐brain exploratory analyses largely recapitulated the findings from our ROI‐based analyses. On the other hand, our TC whole‐brain analyses suggested that even in the absence of navigational demands, the way visual self‐motion cues are processed and/or the brain regions with which this information is shared are related to perceived spatial navigation ability.

Notably, the functional connectivity patterns of OF‐sensitive regions reported to be highly selective for egomotion‐compatible stimuli (L CSv and R CSv) were positively associated with self‐reported navigation ability during both VPI and TC. The only inverse relationship involved the functional connectivity pattern of R pVIP during TC. R pVIP prefers egomotion‐compatible stimuli, but also responds to egomotion‐incompatible stimuli (Cardin & Smith, 2009). Similarly, L/R pV6 respond to both types of stimuli, but we did not find a relationship between the functional connectivity patterns of L/R pV6 during our tasks and self‐reported navigation ability in this experiment. It is possible that these regions play a more fundamental role in processing visual motion cues in navigational contexts because they have been reported to increase their interaction with the hippocampus and retrosplenial cortex during goal directed navigation in first person perspective (Sherrill et al., 2015). It is also possible that they simply were not relevant to the performance of the tasks used in this study. The functional connectivity patterns of L/R pV6 during other types of navigation tasks may be related to perceived spatial navigation ability, and future work should examine this.

There is much to explore in the functional connectivity patterns of OF‐sensitive cortical regions in the context of spatial navigation. The computations that occur in these OF‐sensitive regions and how they relate to the formation of mental representations of space and spatial navigation ability are open questions. It is unclear whether all of these regions contribute to visual motion processing relevant to navigation in all individuals or whether some individuals rely more on certain regions than others. It is also unclear whether any of these regions are absolutely necessary to visual motion processing used in navigational contexts. Studying this will help us to better understand how the brain forms representations of space and what mechanisms form the most accurate or most useful representations. This knowledge could lead to strategies that strengthen navigation ability in a young healthy population, which has the potential to lead to greater preservation of this ability with age and prolonged independence of the aging population.
