# Supplementary material for: Self‐reported navigation ability is associated with optic flow‐sensitive regions’ functional connectivity patterns during visual path integration
Source: Brain Behav. 2019 Mar 18;9(4):e01236. doi: 10.1002/brb3.1236 (PMC6456774; doi:10.1002/brb3.1236)
Supplement: Supplementary file 1 [file BRB3-9-e01236-s001.pdf]

Supporting Information

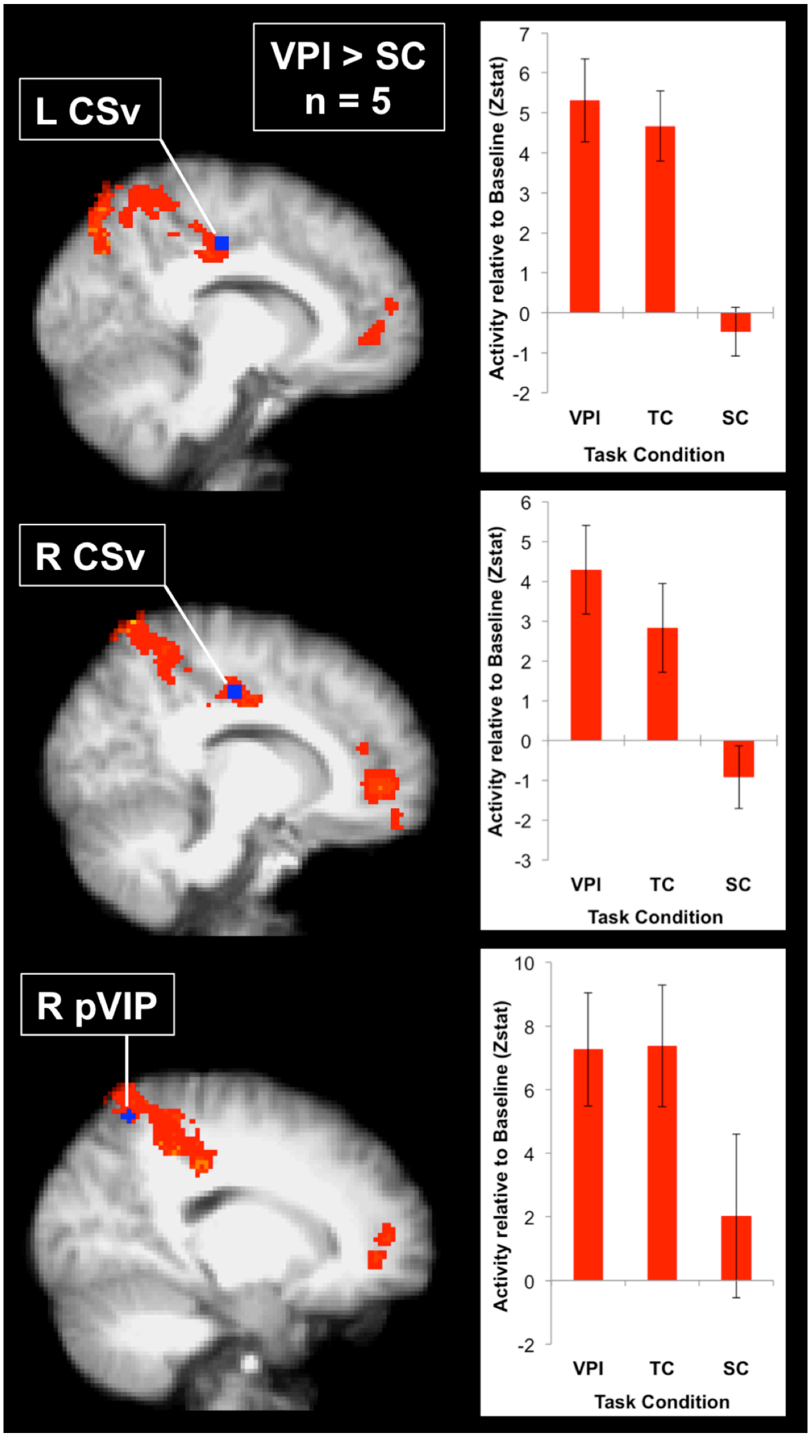

Supplementary Figure 1: Optic Flow-Sensitive Regions Responded More Strongly while Participants Viewed Environmental Stimuli Containing Egomotion Compared to while they Viewed Series of Environmental Stills Containing No Motion. An independent

sample of 5 participants viewed two runs each of three tasks: visual path integration (VPI), turn counting (TC), and still counting (SC). Each run contained 8 trials of each task totaling 16 trials of VPI, TC, and SC. VPI was always shown first while TC and SC conditions were shown second and third and their order was counterbalanced across participants. VPI and TC tasks contained a subset of the stimuli shown to the 15 participants in the main study and the tasks were the same as described in Methods. In the SC task, participants were shown blocks of serially presented still images taken from VPI and TC stimuli that were not shown to them during the VPI and TC runs and they were asked to count the number of images presented in the series. Still blocks were interspersed with rest blocks showing a white fixation cross on a black screen. Stills appeared consecutively within a block (i.e. one after another with no intervening stimulus) and each was shown for 0.5 to 2 seconds. Consecutively presented stills from disparate paths were chosen to ensure that the series of presented stills did not create the perception of self-motion. At the end of each block of stills, a number was presented and participants were asked to select "yes" if the number of stills matched the number of stills they counted in the block. Otherwise, they were asked to select "no". First-level processing was equivalent to the whole-brain processing described in Methods, specifically in the section titled "Defining Target and Control ROIs". A double-gamma HRF convolution was applied to the stimulus waveform representing VPI, TC, or SC in order to model where brain activity increased during VPI, TC, and SC blocks relative to rest. In second-level processing, VPI>SC and TC>SC contrasts were performed to determine where brain activity was greater during VPI or TC, which contained egomotion, compared to SC, which did not contain any motion but contained the same visual characteristics of the stimuli viewed during VPI and TC. FSL's FLAME 1+2 with a Z threshold of 2.3 and a FWER-

corrected cluster p threshold of 0.05 was used in the group-level analysis. The group-level activation maps showing where brain activity was greater during VPI relative to SC are shown on the mean T1 images in MNI152 2mm space of these 5 participants in Supplementary Figure 1. L CSv, R CSv, and R pVIP are shown in blue overlaid on these activity maps. All three OF-sensitive regions overlap with regions showing greater activity during VPI relative to SC (shown) and during TC relative to SC (not shown), suggesting that these OF-sensitive regions are involved in motion processing during VPI and TC. To the right of each activity map, the average Z statistic representing the strength of brain activity in each region during VPI, TC, and SC relative to rest are shown (error bars represent standard error of the mean). Brain activity was significantly greater during VPI vs. SC and during TC vs. SC in all three OF-sensitive regions ($p \leq 0.01$), mirroring their overlap with significantly increased activity on the whole-brain maps. L CSv: left cingulate sulcus visual area, MNI: Montreal Neurological Institute, OF: optic flow, R CSv: right cingulate sulcus visual area, R pVIP: right putative ventral intraparietal area, SC: still counting, TC: turn counting, VPI: visual path integration.
